# Abcès de la cuisse: penser à la tuberculose

**DOI:** 10.11604/pamj.2013.16.32.3244

**Published:** 2013-09-29

**Authors:** Leila Metoui, Faida Ajili

**Affiliations:** 1 Service de médecine interne. Hôpital militaire de Tunis, Tunisie

**Keywords:** Abcès, tuberculose, abscess, tuberculosis

## Image en medicine

La tuberculose se caractérise par la fréquence et le polymorphisme des localisations extra-pulmonaires qui représentent en moyenne 10 à 20% des cas. L'atteinte musculaire est rare, sa prévalence étant évaluée entre 0,01 et 2%. Sa survenue sans lésion osseuse sous jacente, comme c'est le cas de notre patiente, est encore plus rare. Il s'agissait d'une patiente âgée de 32 ans sans antécédents pathologiques notables qui était hospitalisée pour une tuméfaction de la cuisse gauche évoluant depuis 4 mois. L'examen avait objectivé une patiente apyrétique, en bon état général. La face supéro-externe de la cuisse gauche était occupée par une masse fluctuante non inflammatoire, non soufflante, indolore de 19 cm de grand axe. L’échographie des parties molles objectivait une collection sous cutanée bien limitée, hypoéchogéne et hétérogène. L'imagerie par résonance magnétique mettait en évidence la présence d'une volumineuse formation kystique de la face externe de la cuisse gauche sans atteinte osseuse sous-jacente. La scintigraphie osseuse était sans anomalie en dehors d'une hyperfixation au niveau du muscle quadriceps gauche. La biologie trouvait un syndrome inflammatoire avec une VS à 140 mm la première heure, une CRP à 88 mg/l et une fibrinémie à 5,4 g/l sans hyperleucocytose. La sérologie VIH était négative. L'intradermo-réaction (IDR) à la tuberculine était à 5 mm. La mise à plat chirurgicale de cette collection avait objectié un liquide purulent dont l'examen bactériologique a permis d'isoler un Bacille de Koch à la culture. Les autres examens complémentaires (radiographies standards, échographie, examens spécialisés, recherche de BK dans les crachats et les urines,…) n'ont pas permis d'objectiver une autre localisation tuberculeuse. En post opératoire, la patiente a été mise sous quadrithérapie anti-tuberculeuse (Rifampicine, Isoniazide, Ethambutol, Pyrazinamide) pendant 2 mois relayée par une bithérapie par Rifampicine et Isoniazide pendant 10 mois. L’évolution était favorable avec un recul de 6 mois.

**Figure 1:**
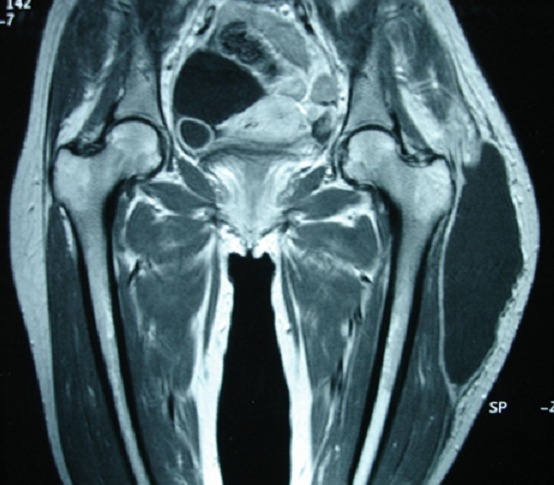
IRM séquence T1 montrant une volumineuse formation kystique en hyposignal T1 de la face supéro-externe de la cuisse gauche sans atteinte osseuse sous jacente

